# Antigen exposure in the late light period induces severe symptoms of food allergy in an OVA-allergic mouse model

**DOI:** 10.1038/srep14424

**Published:** 2015-09-30

**Authors:** Kana Tanabe, Eri Kitagawa, Misaki Wada, Atsushi Haraguchi, Kanami Orihara, Yu Tahara, Atsuhito Nakao, Shigenobu Shibata

**Affiliations:** 1 Department of Physiology and Pharmacology, School of Advanced Science and Engineering, Waseda University, Tokyo, Japan; 2 Waseda Institute for Advanced Study, Waseda University, Tokyo, Japan; 3 Department of Immunology, Faculty of Medicine, University of Yamanashi, Yamanashi, Japan

## Abstract

The mammalian circadian clock controls many physiological processes that include immune responses and allergic reactions. Several studies have investigated the circadian regulation of intestinal permeability and tight junctions known to be affected by cytokines. However, the contribution of circadian clock to food allergy symptoms remains unclear. Therefore, we investigated the role of the circadian clock in determining the severity of food allergies. We prepared an ovalbumin food allergy mouse model, and orally administered ovalbumin either late in the light or late in the dark period under light-dark cycle. The light period group showed higher allergic diarrhea and weight loss than the dark period group. The production of type 2 cytokines, IL-13 and IL-5, from the mesenteric lymph nodes and ovalbumin absorption was higher in the light period group than in the dark period group. Compared to the dark period group, the mRNA expression levels of the tight junction proteins were lower in the light period group. We have demonstrated that increased production of type 2 cytokines and intestinal permeability in the light period induced severe food allergy symptoms. Our results suggest that the time of food antigen intake might affect the determination of the severity of food allergy symptoms.

In mammals, various physiological phenomena, such as thermoregulation and the sleep-wake cycle, are under the control of the circadian clock^1^. The master clock, which generates the circadian rhythm with an approximately 24 hours cycle, is located in the suprachiasmatic nuclei (SCN) of the hypothalamus^2^. Interestingly, circadian oscillators are also present in peripheral tissues^3^, including the lymphoid organs and blood cells^4^. Many functions and parameters of the immune system exhibit circadian rhythms. For instance, the number of lymphocytes in circulation is the highest during the rest period in both diurnal humans and nocturnal rodents^5^,^6^. Not only physiological functions but also several chronic diseases, including allergies, are known to exhibit circadian exacerbations in their symptoms or presentations^7^,^8^,^9^,^10^. In human studies, it has been reported that allergic symptoms, such as those in allergic rhinitis and bronchial asthma, are exacerbated overnight or early in the morning^9^. As the prevalence of allergic rhinitis and asthma grows, the predominance of food allergy is also increasing^11^,^12^. According to self-reported survey data in the United States, within a decade from 1997 there was an 18% increase in food or digestive allergies^12^. Food allergies induce several clinical symptoms systemically and/or locally in the cutaneous, respiratory, and gastrointestinal tracts, such as shock, hives, and diarrhea^12^,^13^. These allergies decrease quality of life and can result in life-threatening reactions.

The food allergy symptoms that are induced within two hours following food ingestion are IgE- and mast cell-mediated reactions^12^. Ingested and digested food proteins are absorbed from the intestinal lumen^14^. This absorption is regulated by intestinal permeability, which is a critical feature of the gastrointestinal epithelium. Permeability allows the efficient absorption of nutrients, and restricts the entry of large molecules so that the appropriate immune responses can occur against food antigens^15^. However, a small proportion of intact antigens can reach the lamina propria, the intestinal lymphatic system, and subsequently join the circulation. These antigens are taken up by antigen-presenting cells in the lamina propria, and then processed and presented to the surrounding T cells or the T cells in mesenteric lymph nodes (MLN)^15^. Recently, several studies have investigated the circadian regulation of intestinal permeability and tight junctions (TJs) which make up the paracellular pathway^16^,^17^,^18^. Further, TJs are known to be affected by cytokines^19^.

Type 2 cells that are presented food antigens produce type 2 cytokines, such as IL-4, IL-5, and IL-13, in response. The production of type 2 cytokines is controlled by several transcription factors, including STAT6, GATA3, NFAT, NF-κB, c-Maf, AP-1, and E4BP4^20^,^21^,^22^. In particular, it has been evidenced that E4BP4 is directly controlled by clock genes and that its expression level in human CD4^+^ T-cells follow circadian rhythm^5^,^23^. IL-13 induces IgE class switching in human B cells, leading to an allergic reaction^24^. IgE binds to mast cells; upon re-exposure to antigenic proteins, antigens bind to and cross-link these cell surface-bound IgE molecules, inducing the release of symptom-causing mediators, such as histamine and serotonin^25^. The expression of the high-affinity IgE receptor (FcεRI) on mast cells is also regulated by the intrinsic clock of the cells^26^.

As the contribution of the circadian clock in the development of food allergy symptoms remains unknown, we hypothesized that the circadian clock plays a role in determining the severity of food allergy symptoms. Therefore, in this study, we prepared an ovalbumin (OVA) food allergy mouse model, and investigated whether the severity of food allergic reactions differed based on the timing of OVA oral administration.

## Results

### Food allergic diarrhea was severe in the light period

**Figure 1:**
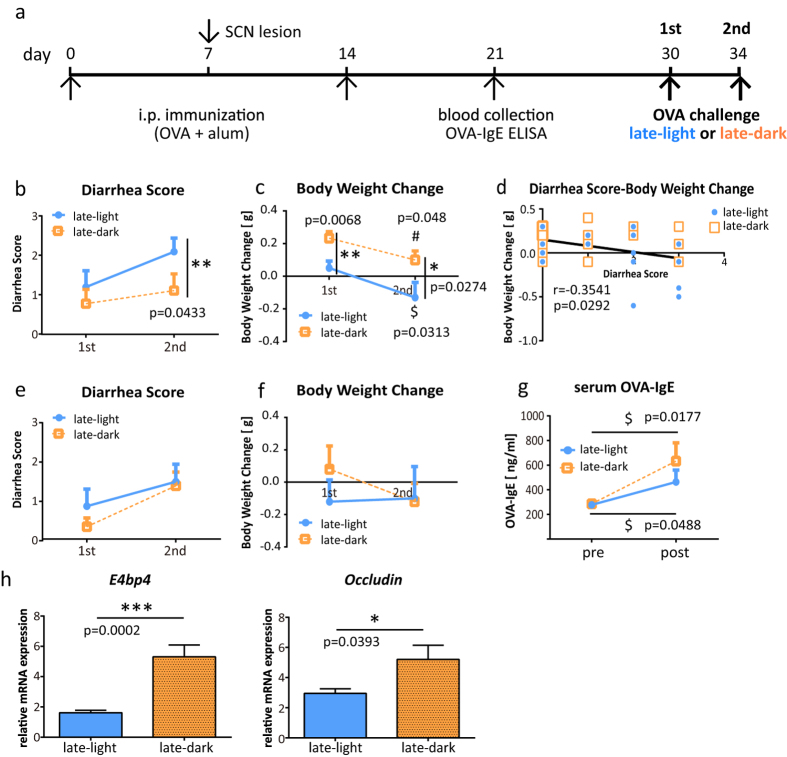
Mice orally administered ovalbumin (OVA) in the light period had more severe local allergic reactions. (**a**) Immunization schedule for the experiment. Female intact or SCN lesioned BALB/c mice were sensitized and subsequently treated with orally administered OVA (80 mg/500 μl distilled water) in the light period or the dark period. (**b**) The diarrhea score was assessed 30–60 min after treatment in intact mice. (**c**) Body weight was measured before and after treatment in intact mice. The pre-treatment body weight was subtracted from the post-treatment body weight. (**d**) Significant correlation between the diarrhea score and change in body weight. The data from each individual in the first and the second challenges are plotted. *p < 0.05. Spearman’s rank correlation coefficient was calculated to assess correlation. (**e**) The diarrhea score was assessed 30–60 min after treatment in SCN lesion mice. (**f**) Body weight was measured before and after treatment in SCN lesion mice. The pre-treatment body weight was subtracted from the post-treatment body weight. The data (**e**,**f**) are presented as the means ± SEM (n = 10). (**g**) An increase in OVA-specific serum levels of IgE, in intact mice measured by ELISA. (**h**) The mRNA expression levels in the jejunum were measured using real-time RT-PCR. The data (**b**,**c**,**g**) are presented as the means ± SEM (n = 9–10). Data (**b**,**c**) *p < 0.05, **p < 0.01 in the light period versus the dark period calculated with a t-test. $p < 0.05 for the light period of first challenge versus the light period of second, calculated with a Wilcoxon signed-rank test. #p < 0.05 for the dark period of the first challenge versus the dark period of the second challenge, calculated with a t-test. Data (**g**) $p < 0.05 pre-treatment versus post-treatment calculated with a Wilcoxon signed-rank test. The data (**h**) are presented as means ± SEM (n = 7–10). ***p < 0.001, *p < 0.05 for light period versus dark period, calculated with a Mann-Whitney test.

We investigated whether the symptoms of food allergies differed in severity when the time of the oral treatment was changed. Mice were orally administered OVA in the late light period (light period group) or in the late dark period (dark period group). The overall diarrhea score was higher in the light period group than the dark period group in both the first and the second challenges (Fig. 1b). Accompanied with the diarrhea, most of the mice lost weight. The light period group lost significantly more weight than the dark period group (Fig. 1c). Furthermore, the diarrhea score showed a negative correlation with body weight change (Fig. 1d). To investigate the link between the circadian clock and food allergy, OVA sensitized SCN lesioned mice^27^ as a circadian disruption mouse model were challenged with OVA. OVA-specific serum IgE before OVA challenge in SCN lesioned mice was comparable with that in intact mice (data not shown). Significant differences of diarrhea score and body weight change that were seen between the light period and the dark period groups disappeared when mice had lesioned SCN (Fig. 1e,f). This result suggests that SCN, which generates the circadian rhythm, regulates the severity of food allergy symptoms, and that the timing of food-antigen intake is one of the factors that determine the severity of food allergic diarrhea.

### OVA-specific serum IgE increased following OVA oral treatment

To confirm that the oral OVA treatment induced an increase in OVA-specific serum IgE, mice were sacrificed 30–60 minutes after the second challenge, and blood samples were collected. Serum OVA-IgE levels were significantly higher after the treatment compared to the levels before the treatment, both in the light period and the dark period groups (Fig. 1g). However, the post-treatment OVA-IgE levels showed no significant difference between the light period and dark period groups.

### mRNA expression of transcription factor *E4BP4* and TJ protein *Occludin* was lower in the light period than in the dark period

The mRNA levels of the type 2 cytokine transcription factor *E4BP4* and of the TJ protein *Occludin* (*Ocln*) were examined in the jejunum tissue obtained from the mice that received oral treatment twice. The mRNA expression levels of *E4BP4* were found to be significantly lower in the light period group than in the dark period group (Fig. 1h). Similarly, the expression levels of *Ocln* were also lower in the light period group (Fig. 1h).

### The production of IL-13 and IL-5 by MLN cells was higher in the light period

**Figure 2:**
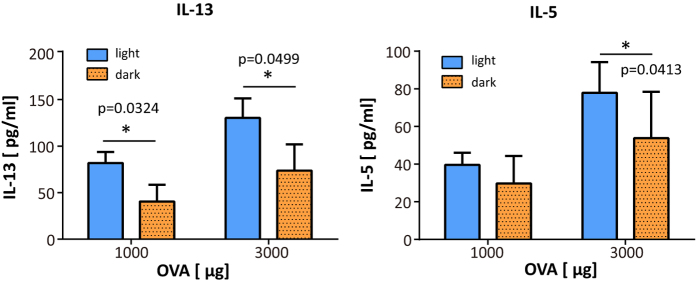
Mesenteric lymph node (MLN) cells from the light period ovalbumin (OVA) treated group produced higher IL-13 and IL-5 levels than the dark period treated group. After the second treatment, MLN cells were isolated and incubated with 1000 or 3000 μg of OVA. The type 2 cytokines IL-13 and IL-5 were measured in the culture medium by ELISA. The data are presented as means ± SEM. (n = 8). *p < 0.05 for light period treatment versus dark period treatment, calculated with a Mann-Whitney test.

We investigated the response of immune cells that were stimulated at different time points with OVA antigen. After the second challenge, MLN cells were harvested and stimulated with OVA *in vitro*. After three days, the culture supernatants were collected and type 2 cytokine production levels were measured. Cells obtained from the light period group produced significantly higher levels of IL-13 than the dark period group (Fig. 2). Although the data for IL-4 were below the detection limit, similar results of differential production level were obtained for IL-5 (Fig. 2). These results demonstrate that antigen stimulation in the light period, as opposed to the dark period, induces higher IL-13 and IL-5 production.

### The absorption of orally administrated OVA into the blood was greater in the light period than the dark period

**Figure 3:**
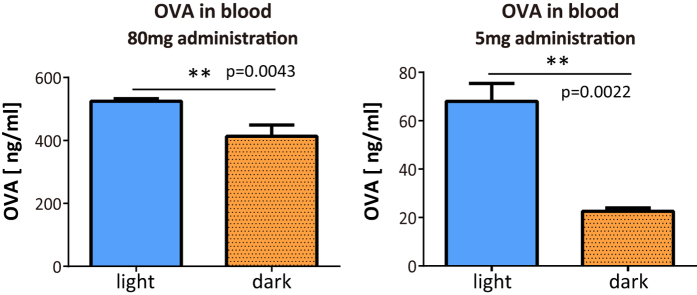
A higher concentration of ovalbumin (OVA) was absorbed into the blood in the light period treatment group than the dark period group. Non-sensitized mice were administrated OVA orally (80 mg/500 μl or 5 mg/300 μl distilled water) in the light period or in the dark period. Blood samples were collected after 30 min. The serum OVA level was measured by ELISA. The data are presented as means ± SEM. (n = 5–6). **p < 0.01 light period treatment versus dark period treatment, calculated with a Mann-Whitney test.

We found a significant difference between the food allergic diarrhea induced in the light period and that in the dark period. We therefore hypothesized that OVA absorption is different in the light period and the dark period. In the body, the proteins that are absorbed from the intestinal lumen migrate to the blood stream. Thus, to determine OVA absorption, we measured the OVA concentration in the blood following oral OVA administration. Blood samples were collected 30 minutes after OVA administration in the light period or in the dark period. It has been well demonstrated that the OVA concentration in the blood reaches peak levels 20–30 minutes after oral administration^28^. We found that there were significant differences in the serum OVA levels at both oral doses used. Higher concentrations of OVA were absorbed in the light period group than the dark period group (Fig. 3).

### Intestinal permeability was higher in the light period than in the dark period

**Figure 4:**
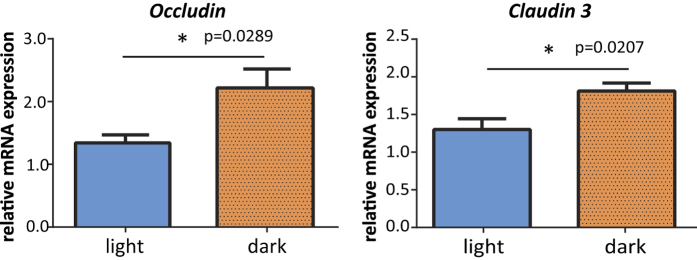
Tight junction (TJ) mRNA expression levels of *Occludin* (*Ocln*) and *Claudin 3* (*Cldn3*). Jejunum tissues were collected from BALB/c mice that were sensitized but were not given any oral challenge with ovalbumin in the light period or the dark period. The mRNA expression levels of the TJ proteins *Ocln* and *Cldn3* were measured using real time RT-PCR. The data are presented as means ± SEM (n = 5). *p < 0.05 light period group versus dark period group, calculated with a t-test.

We investigated whether the intestinal permeability in the mice was different in the light period and the dark period. We quantified the mRNA expression levels of TJ proteins, *Ocln* and *Claudin 3* (*Cldn3*), in the jejunum. The mRNA expression levels of these genes were significantly lower in the light period group than the dark period group (Fig. 4). This result suggests that the intestinal permeability may be higher in the light period than the dark period.

## Discussion

In this study, we investigated whether the circadian clock regulates the severity of food allergy symptoms. Here, we demonstrated that an allergen intake late in the light period induces severe allergic diarrhea, greater than that observed upon allergen ingestion in the dark period, in the OVA food allergy model mice. We conducted the same experiment in SCN-lesioned mice, as a mouse model of circadian clock disruption. Significant differences that were seen between the light period and the dark period groups in the present study disappeared when mice had lesioned SCN. This result suggests that SCN, which generates the circadian rhythm, regulates the severity of food allergy symptoms, and that the timing of food antigen intake has an effect upon this severity.

The symptoms that develop rapidly following food ingestion, such as shock and diarrhea, are IgE- and mast cell–mediated immediate-type hypersensitivity reactions^25^,^29^,^30^. For this reason, we measured the serum OVA-specific IgE levels before and after OVA administration. Although there was no significant difference between the light period and the dark period groups, we detected an increase in OVA-specific IgE levels following the OVA administration. Our previous study reported that the mast cell-intrinsic clockwork primarily contributes to the temporal regulation of allergic reactions by regulating the expression of FcεRI in mast cells^26^. In that study, we showed that IgE/mast cell-mediated allergic reaction (passive cutaneous anaphylactic (PCA) reaction) was promoted at the end of the light period. According to the previous and the present results, we hypothesized that the expression levels of FcεRI as well as mast cell degranulation might be increased in the light period. Thus, we measured mRNA expression levels of FcεRI in jejunum tissue that were collected after the second OVA treatment, however, no significant differences were detected (Supplementary Fig. S1). We also measured the intestinal serotonin levels (Supplementary Fig. S2), since serotonin is a key mediator to induce allergic diarrhea^29^. As expected, serotonin levels were higher in the light period group than the dark period group, however this result was not significant. Although the serum OVA-specific IgE levels were not significantly different between the light period and the dark period OVA administered groups, increased production of IL-13 and IL-5 was found in the MLN cell culture supernatant obtained from the light period group. We confirmed that this difference is partly due to the time taken to collect MLN cells and antigen stimulation by performing the same culture experiment in sensitized mice that were not challenged. Although there were no statistically significant differences, the production of IL-13 and IL-5 was also higher in MLN cell culture supernatants obtained from the light period group (Supplementary Fig. S3). Both IL-13 and IL-5 contribute to type 2 inflammation. The observation that different timing of antigen exposure led to a significant difference in the production of IL-13 and IL-5 suggests that this production is somehow regulated by the circadian clock. The expression levels of type 2 cytokines are controlled by several transcription factors. As E4BP4 is directly controlled by clock genes^5^, we focused on this transcription factor. E4BP4 is also known to suppress the expression of IL-13 and IL-5, and directly binds to and negatively regulates the gene expression of *Il-13*^22^. Our data demonstrated that mRNA expression level of *E4BP4* in the jejunum tissue of the light period group was significantly lower than that in the dark period group. mRNA expression of *E4BP4* in the jejunum obtained from mice that were not challenged was also significantly different; the light period group showed lower expression (Supplementary Fig. S4). Thus, lower expression level of *E4BP4* in the light period seems to be one of the factors that promote IL-13 and IL-5 production. In contrast, E4BP4 appears to promote IL-4 expression indirectly^22^. Although our IL-4 data in MLN cell culture supernatants were below the detection limit, the production of IL-4 may be postulated to be higher in the dark period group, due to the higher expression of *E4BP4*. IL-4 regulates immunoglobulin class switching to IgE in B cells^31^. Thus, higher expression of IL-4 may promote the IgE production seen in the dark period group. This might be one reason why there was no significant difference in the serum OVA-IgE levels between the groups.

Type 2 cytokines also influence intestinal permeability, i.e. the intestinal barrier function^19^. The intestinal permeability regulates protein absorption and affects food allergy symptoms^15^. It is known that the antigens absorbed in the intestine, and not those in the lumen, induce allergic diarrhea^32^. The antigens that cross the epithelial barrier are mostly taken up by the antigen-presenting cells in the lamina propria, and are then processed and presented to the surrounding T cells^15^. This suggests that the higher the amount of antigen being absorbed, the higher T cells proliferate. Intestinal permeability is regulated by the paracellular and transcellular pathways^18^,^33^. Recently, it has been revealed that OVA (45 kDa) is absorbed via both the paracellular and transcellular pathways^34^. In particular, TJs, which make up the paracellular pathway, are known to be affected by cytokines^19^. Previous studies have reported that IL-13 decreases the expression of Occludin^35^, and also increases paracellular permeability in colonic epithelial cells^36^. Our study showed that the mRNA expression of *Ocln* in the jejunum was significantly lower when OVA was ingested in the light period than the dark period. Taken together, these results suggest that the higher levels of IL-13 production in the light period group may decrease the expression of *Ocln*, leading to increased paracellular permeability. A previous study reported that the expression of *Ocln* and *Claudin 1* is under circadian control in the murine large intestine^17^. According to this study, mRNA expression levels of these genes are higher in the middle of the light period and lower in the middle of the dark period. Consistent with mRNA expression, the protein expression levels in the middle of the light period are higher than in the dark period. There might be a time-lag between mRNA and protein expression, however, it seems that in this case the protein and mRNA expression do not cycle in the opposite phases of the circadian cycle. The circadian expression of TJ proteins was associated with temporal changes in colonic permeability and also with susceptibility to colitis. In agreement with this study, it seems that the increased paracellular permeability observed in the light period group in our study promotes OVA absorption and type 2 immune responses at this time of day in the cycle.

To confirm whether there is a difference in OVA absorption between the light period and the dark period, we compared the serum OVA concentration in the light period and the dark period groups. For both doses (5 or 80 mg), the OVA concentration in the serum was significantly higher in the light period group, therefore the absorption of OVA is greater in the light period group. We also compared the mRNA expression levels of *Ocln* and *Cldn3*. Claudin 3 is a known TJ protein and is highly expressed in the jejunum^37^. Our data showed that both the mRNA expression levels of *Ocln* and those of *Cldn3* were lower in the light period group. Taken together, these results suggest that the lower expression of TJ proteins in the light period group increases paracellular permeability and, perhaps as a result, the absorption of OVA is facilitated. We hypothesized that the increased paracellular permeability also facilitates the passage of other proteins, and thus we performed the same experiments with the food antigen, β-lactoglobulin (18 kDa). In accordance with our expectations, β-lactoglobulin tended to be absorbed at a higher concentration in the light period group than in the dark period group, although this result was not statistically significant (Supplementary Fig. S5). As we mentioned above, OVA can be absorbed via both the paracellular and transcellular pathways. However, the mechanism underlying transcellular absorption is not fully understood. Our data suggest that OVA absorption in the transcellular pathway may also be regulated by the circadian clock.

Overall, type 2 cytokine production seems to be dependent on the expression of *E4BP4*. The expression levels of *E4BP4* are lower in the light period group, which seems to lead to the greater IL-13 and IL-5 production and to lower production of IL-4 in the light period group when compared to the dark period group. In addition, the type 2 immune response seemed to be promoted in the light period group due to the higher amount of OVA absorption. This appears to be due to leakage in the absorption in the paracellular pathway. In our study, the serum OVA-specific IgE levels were not different between the light period and the dark period group. However, a previous study has reported that food allergy patients showed increased intestinal permeability, which was significantly correlated with the severity of their clinical symptoms^38^. This perfectly aligns with our findings. Thus, it is proposed that increased intestinal permeability may directly contribute to allergic symptoms. For these reasons, food allergy symptoms may be exacerbated in the light period.

In summary, this is the first study to investigate whether the circadian clock plays a role in food allergies. We have demonstrated that food antigen exposure in the light period induces more severe food allergic symptoms than exposure in the dark period, in an OVA allergic mouse model. This suggests that the timing of food intake may affect the determination of the severity of food allergy symptoms.

## Methods

### Mice and SCN lesion

Female BALB/c mice were purchased from Tokyo Laboratory Animals Science CO. LTD (Tokyo, Japan). The mice were housed at 22 °C with a humidity of approximately 60% under a 12 hour light/dark cycle. The lights were turned on at 08:00 and turned off at 20:00. Mice were fed a normal commercial diet (Catalog #MF; Oriental Yeast Co., Ltd., Tokyo, Japan) and were given *ad libitum* access to water. To investigate the role of SCN on time-dependent difference of OVA-induced food allergy, SCN was lesioned in some mice. As described in our previous paper^27^, bilateral thermal lesion of the SCN was performed stereotaxically, and locomotor arrhythmicity and histological SCN lesion sites were verified (N = 20). The procedures conformed to the “Fundamental Guidelines for Proper Conduct of Animal Experiment and Related Activities in Academic Research Institutions” (published by the Ministry of Education, Culture, Sports, Science and Technology, Japan) and were approved by the Committee for Animal Experimentation of the School of Science and Engineering at Waseda University (permission #2013-A064).

### OVA sensitization and challenge

Mice (6–7 weeks old) were sensitized with 10 μg of OVA (Sigma-Aldrich, St. Louis, MO, U.S.A.) and 1 mg of aluminum (Wako Pure Chemical Industries, Ltd, Osaka, Japan) in Hyper-Pure water by intraperitoneal injection twice at two-week intervals. To check whether OVA-specific IgE was produced by sensitization, peripheral blood was collected, under isoflurane anesthesia, from the orbital veins a week after the second sensitization event, using capillary glass tubes. Then, the level of serum OVA-specific IgE was measured by ELISA (DS Pharma Biomedical Co., Ltd., Osaka, Japan).The assay was performed according to the manufacturer’s instructions.

Ten days after the blood collection, mice were orally administrated OVA at a dose of 80 mg/mouse, dissolved in 500 μl distilled water, either at 05:00 or 17:00. Since mice are nocturnal, 05:00 corresponds to the late dark period and 17:00 corresponds to the late light period. Prior to administration, mice were fasted for six hours. The mice were treated twice with OVA, and the second treatment was performed three days after the first treatment. As the second treatment induced more severe symptoms than the first, we hypothesized that the effect of the different times at which the food antigen was administered would be observed more obviously. The immunization schedule is shown in Fig. 1a. and in Supplementary Fig. S6.

### Evaluation of food allergy symptoms

Food allergy symptoms were evaluated with the parameters of allergic diarrhea and change in body weight. Allergic diarrhea was apparent 30–60 minutes following the treatment, and it was assessed by a severity score of the fecal form from 0 to 3: 0, no fecal matter or solid state; 1, funicular form; 2, slurry; 3, watery state, as used in previous papers^39^. The criterion for scoring is shown in Supplementary Fig. S7. Body weight of the mice was measured prior to the treatment and also when the diarrhea score was assessed. 30–60 minutes after OVA administration, blood samples were collected to measure the level of serum OVA-specific IgE.

### Preparation of single-cell suspensions of MLN

After the second evaluation of food allergy symptoms or without OVA administration, MLN were immediately removed and cell suspensions were prepared. MLN were put into a petri dish containing 10 ml of RPMI1640 medium (Sigma-Aldrich) and were then crushed with a microscope slide to tear the capsule. The cell suspension was passed through a 70 μm cell strainer into a 50 ml tube, and then centrifuged at 4 °C at 1200 rpm for 5 minutes. After centrifugation, the supernatant was discarded. One milliliter of ACK buffer (Lonza, Tokyo, Japan) was added to the tube and the tube was then incubated on ice for 3 minutes. Immediately after that, 50 ml of RPMI medium was added and the solution was centrifuged. The supernatant was discarded and 5 ml of RPMI medium was added. The resulting solution was a single-cell suspension of MLN.

### Cytokine production

Cells were suspended in RPMI medium to achieve a final concentration of 1.3 × 10^6^ cells/ml. They were then incubated with OVA (1000 or 3000 μg/ml) for 72 hours. Subsequently, the supernatant was collected by centrifugation. IL-13 and IL-5 levels in the culture supernatants were measured by ELISA (R&D Systems, Minneapolis, MN, USA). The assays were performed according to the manufacturers’ instructions.

### Isolation of total RNA and Real-time RT-PCR

Jejunum tissue was collected from mice sacrificed either before or after the challenge with OVA. Total RNA was extracted with phenol. A 50 ng aliquot of total RNA was reverse-transcribed and amplified with a One-Step SYBR reverse transcription PCR (RT-PCR) kit (TaKaRa Bio Inc, Shiga, Japan) in the PikoReal Real-Time PCR System (Thermo Fisher Scientific, Inc., Kanagawa, Japan). The specific primer pairs were designed based on published data for *18S ribosomal RNA (rRNA)*, *Ocln*, *Cldn3*, and *E4bp4* genes^40^,^41^,^42^. The primer sequences are described in Supplementary Table. S1. The relative level of the target gene PCR product was normalized to that of *18s rRNA*. The data were analyzed using the ΔΔCt method.

### Absorption of orally-administrated OVA into blood

Non-sensitized mice (10–12 weeks old) were fasted for six hours prior to administration. Mice were administered OVA orally at a dose of either 80 mg/500 μl or 5 mg/300 μl distilled water in the light period or the dark period. Blood samples were collected 30 min after this administration. Using these blood samples, the OVA concentration in the serum was determined. The OVA concentration was measured by ELISA (Institute of Tokyo Environmental Allergy, Inc., Tokyo, Japan), performed according to the manufacturer’s instructions.

### Statistical analysis

Statistical analyses were performed using GraphPad Prism software, version 6.03 (GraphPad Software, San Diego, CA, USA). To check whether the data was normally distributed and showed equal or biased variance, we used the D’Agostino–Pearson test/Kolmogorov–Smirnov test and calculated the F value. Parametric analysis was conducted using a Student’s t-test. Non-parametric analysis was done using a Mann–Whitney test and a Wilcoxon signed-rank test. The data are presented as means ± SEM. The value p < 0.05 was considered significant.

## Additional Information

**How to cite this article**: Tanabe, K. *et al.* Antigen exposure in the late light period induces severe symptoms of food allergy in an OVA-allergic mouse model. *Sci. Rep.* **5**, 14424; doi: 10.1038/srep14424 (2015).

## Supplementary Material

Supplementary Information
