# Supplementary material for: Antigen exposure in the late light period induces severe symptoms of food allergy in an OVA-allergic mouse model
Source: Sci Rep. 2015 Sep 30;5:14424. doi: 10.1038/srep14424 (PMC4588575; doi:10.1038/srep14424)
Supplement: Supplementary Information [file srep14424-s1.pdf]

Supplementary Information for

Antigen exposure in the late light period induces severe symptoms of food allergy in an OVA-allergic mouse model

Kana Tanabe, Eri Kitagawa, Misaki Wada, Atsushi Haraguchi, Kanami Orihara, Yu Tahara, Atsuhito Nakao, and Shigenobu Shibata

This PDF file includes
Figs. S1 to S7
Table. S1

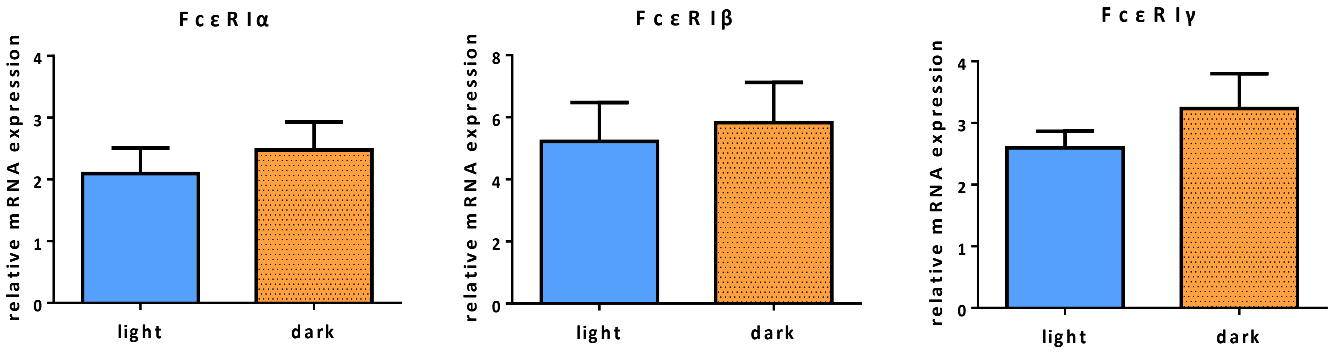

Supplementary Figure. S1 mRNA expression levels of FcεRI.

Jejunum tissues were collected after the second challenge. The mRNA expression levels of FcεRI were measured using real time RT-PCR. The specific primer pairs were designed with Primer3 Input software (version 0.4.0; Whitehead Institute for Biomedical Research, Massachusetts, U.S.A.). Data are presented as means ± SEM (n=7-10).

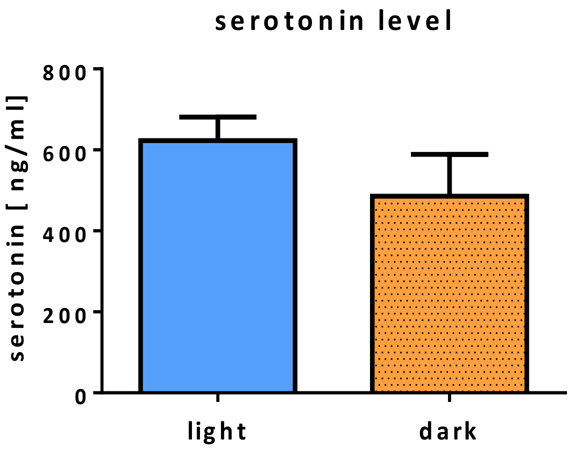

Supplementary Figure. S2 Intestinal serotonin concentration.

Jejunum tissues that were collected after the second challenge were put into a tube containing 500 μl PBS and protease inhibitor (Roche Diagnostics, Indiana, U.S.A.). Jejunum tissues were prepared as tissue homogenates, and centrifuged. The supernatant was used for measuring serotonin level by ELISA (Enzo Life Sciences, NY, U.S.A.) and total protein levels by BCA Protein Assay kit (Thermo Fisher Scientific, Massachusetts, U.S.A.). Serotonin level was normalized to total protein levels. Data is presented as means ± SEM (n=5).

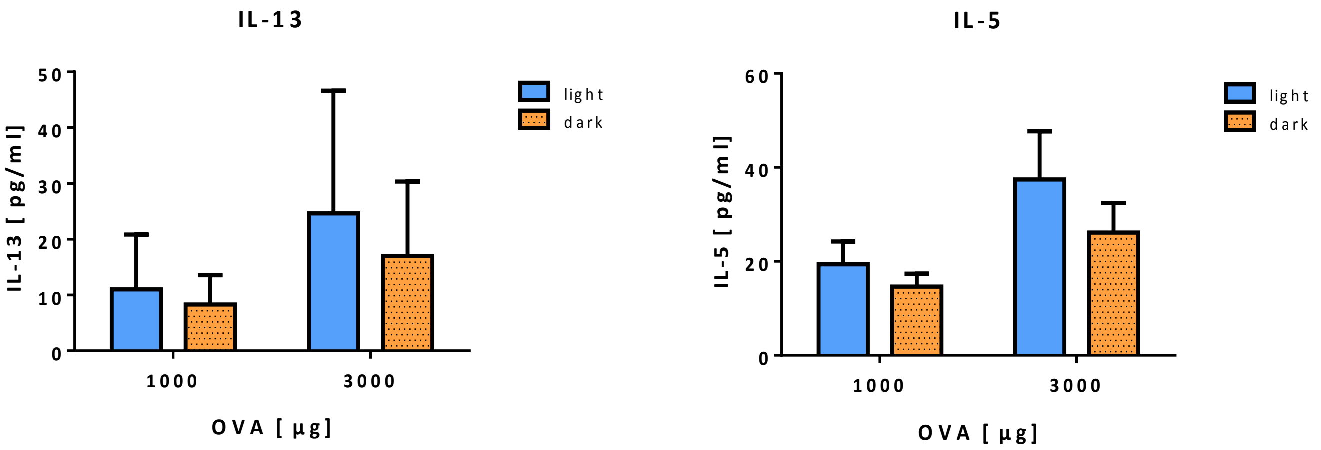

Supplementary Figure. S3 The production of IL-13 and IL-5 from MLN cells obtained from non-challenged mice.

MLN were collected from non-challenged mice at the late light or the late dark period. MLN cells were isolated, and incubated with OVA for 72 hours. The levels of IL-13 and IL-5 in culture supernatants were measured by ELISA. Data are presented as the means ± SEM (n=5).

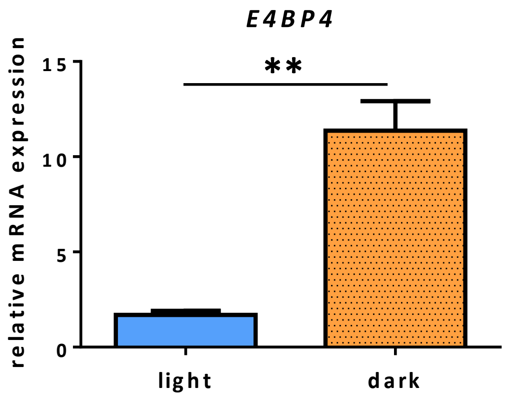

Supplementary Figure. S4 mRNA expression level of *E4bp4*.

Jejunum tissues were collected from non-challenged mice at the late light or the late dark period. The mRNA expression level of *E4bp4* was measured using real time RT-PCR. Data is presented as the means ± SEM (n=5).

**p<0.01 light versus dark, calculated with a Mann-Whitney test.

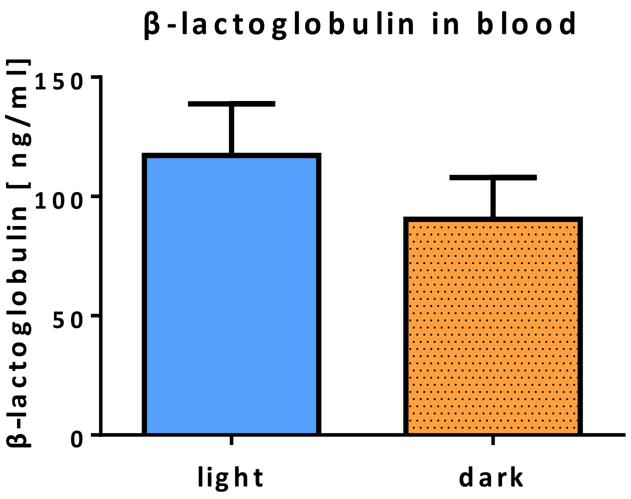

Supplementary Figure. S5 Absorption of β-lactoglobulin into blood.

Non-sensitized mice were orally administrated β-lactoglobulin (50mg / 500μl distilled water) at the late light or the late dark period. Blood samples were collected after 30 minutes. Serum β-lactoglobulin level was measured by ELISA (Morinaga Institute of Biological Science, Inc, Kanagawa, Japan). Data is presented as means ± SEM (n=5).

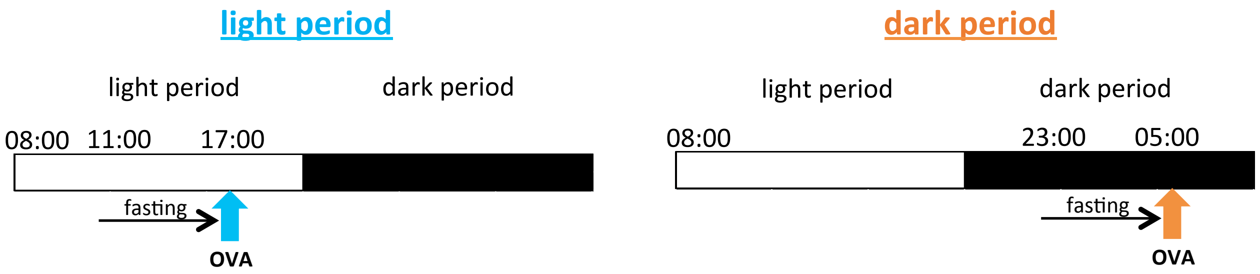

Supplementary Figure. S6 Schedule for oral OVA administration.

Left: light period group Right: dark period group. Mice in the light period group were orally administrated OVA at 17:00. Mice in the dark period group were orally administrated OVA at 05:00. Mice were fasted 6 hours before administration.

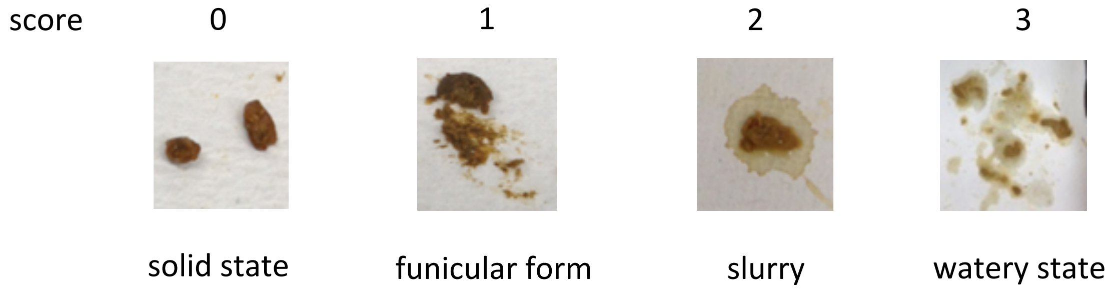

Supplementary Figure. S7 Criterion for allergic diarrhea.

Supplementary Table. S1 Primer sequences.

gene name	Forward sequence (5' → 3')	Reverse sequence (5' → 3')	Source
18s rRNA	ggg gag tat ggt tgc aaa gc	tgt caa tcc tgt ccg tgt cc	40
Ocln	atg tcc ggc cga tgc tct c	ttt ggc tgc tct tgg gtc tgt at	41
Cldn3	cag ggg cag tct ctg tgc gag	gcc gct gga cct ggg aat caa c	41
E4bp4	atg gga agc tct ttc tcc act	tac ccg agg ttc cat gtt tc	42
FcεRIα	atg gtc act gga agg tct gc	tgt tcc cat agc agg aaa gg	This study
FcεRIβ	gct gct ttg tgg ctt ctt tc	caa cac agc act gca aaa gg	This study
FcεRIγ	agc cgt gat ctt gtt ctt gc	ttt cgg acc tgg atc ttg ag	This study
